# Prevalence of Antibiotic Resistance Bacteria in Manure, Soil, and Vegetables in Urban Blantyre, Malawi, from a Farm-to-Fork Perspective

**DOI:** 10.3390/ijerph22081273

**Published:** 2025-08-14

**Authors:** Amon Abraham, Andrew G. Mtewa, Chimwemwe Chiutula, Richard Lizwe Steven Mvula, Alfred Maluwa, Fasil Ejigu Eregno, John Njalam’mano

**Affiliations:** 1 Department of Energy Resources, Ndata School of Climate and Earth Sciences, Malawi University of Science and Technology, Limbe P.O. Box 5196, Malawi; moh-013-22@must.ac.mw; 2 Department of Applied Studies, Chemistry Section, Malawi Institute of Technology, Malawi University of Science and Technology, Limbe P.O. Box 5196, Malawi; amtewa@must.ac.mw; 3 Department of Earth Sciences, Ndata School of Climate and Earth Sciences, Malawi University of Science and Technology, Limbe P.O. Box 5196, Malawi; poh-004-22@must.ac.mw; 4 Directorate of Research and Outreach, Malawi University of Science and Technology, Limbe P.O. Box 2109, Malawi; aomaluwa@must.ac.mw; 5 Department of Building, Energy and Material Technology, Faculty of Engineering Science and Technology, UiT, The Arctic University of Norway, Postboks 385, 8514 Narvik, Norway; fasil.e.eregno@uit.no; 6 Department of Water Resources Management, Ndata School of Climate and Earth Sciences, Malawi University of Science and Technology, Limbe P.O. Box 5196, Malawi; jnjalammano@must.ac.mw

**Keywords:** antibiotics, antimicrobial resistance, *E. coli*, *K. pneumoniae*, manure, One Health, vegetables, zoonotic

## Abstract

The use of untreated livestock manure in urban agriculture sustains soil fertility but risks disseminating antimicrobial resistance (AMR) in resource-limited settings. This study characterized antibiotic-resistant bacteria (ARB) prevalence across manure–soil–vegetable pathways in Blantyre, Malawi. Using a cross-sectional design, we collected 35 samples (poultry/pig manure, farm/home soils, *Brassica rapa* subsp. *chinensis*, *Brassica rapa*, and *Amaranthus* spp.) from five livestock farms. Microbiological analysis with API 20E identification and disk diffusion testing revealed clear differences in contamination: *Escherichia coli* dominated pig manure (52%) and farm soil (35%), with detection in vegetables suggesting possible transfer (e.g., 20% in *Brassica rapa* subsp. *chinensis*), while *Klebsiella pneumoniae* contaminated all sample types (peak: 60% vegetables and 67% home soils). All manure isolates exhibited sulfamethoxazole–trimethoprim resistance, with 50% of pig manure *E. coli* showing cefotaxime resistance. Soil isolates mirrored these patterns (100% ampicillin resistance in *K. pneumoniae* and 77% cefotaxime resistance in farm soil *E. coli*). Vegetables displayed severe multidrug resistance (100% *E. coli* and 80% *K. pneumoniae* resistant to ≥3 classes), including critical gentamicin resistance (100% *E. coli*). Composting for ≤6 weeks, as practiced on the studied farms, did not eliminate ARBs, suggesting that longer durations may be needed. Notably, this study provides the first phenotypic evidence of presumptive *Pasteurella*-like organisms on edible leafy vegetables, specifically 45% in *Amaranthus* spp. and 6.1% in *Brassica rapa*, suggesting a potential zoonotic transmission route from livestock farms that requires molecular confirmation. These findings demonstrate manure-amended farms as AMR reservoirs, necessitating extended composting and antibiotic stewardship to mitigate One Health risks.

## 1. Introduction

The emergence of antimicrobial resistance (AMR) poses a critical threat to global public health, food security, and sustainable development. AMR is linked to 4.95 million human fatalities annually, with a projected annual economic loss of USD 100 trillion by 2050 if not curbed [1]. This represents 27.3 fatalities per 100,000 individuals in sub-Saharan Africa [1]. The six predominant bacteria responsible for mortality linked to AMR in sub-Saharan Africa were found to be *Streptococcus pneumoniae*, *K. pneumoniae*, *E. coli*, *Staphylococcus aureus*, *Pseudomonas aeruginosa*, and *Acinetobacter baumannii* [1]. A surge in AMR has been linked to poor clinical practices, negative public attitudes, and an increase in adverse agricultural practices [2].

One important transmission pathway of AMR that is overlooked is wildlife. Wildlife can work as a sentinel for ARBs and provides an essential domain for the interaction of animals and their environment [3]. The spreading of ARBs in urban environments often involves particular wildlife species that have become accustomed to residing in or near human-made structures for a portion of their life cycles. Additionally, changes of wildlife habitats due to human activities usually force animals to interact more closely with humans, increasing the potential link between ARBs and wildlife. Clinically significant antimicrobial-resistant bacteria have been recovered from many wildlife species, predominantly synanthropic birds, across all continents [4]. This represents a noteworthy one-health issue, since wildlife can easily disseminate ARBs due to their unrestricted movement across countries and continents. There are a few studies on wildlife and the transmission of ARBs [4]. Similarly, in Malawi, few studies are addressing this problem, with most research focusing on ARBs in livestock [4,5,6,7].

Farming practices, particularly the use of livestock manure as fertilizer, are increasingly recognized as key drivers of environmental AMR transmission [8,9]. Studies globally have detected multidrug-resistant pathogens such as *E. coli*, *Salmonella* spp., and extended-spectrum β-lactamase (ESBL)-producing bacteria that persist in soils and contaminate food crops [10,11], with resistance patterns that mirror the antibiotics commonly used in livestock [12]. It is reported that 76% of antibiotic contamination in soils globally originates from untreated manure and wastewater [13,14]. The reliance on untreated manure such as from poultry and pigs, often loaded with tetracycline, sulfonamide, and fluoroquinolone residues [15,16], amplifies the risks of ARB contamination in soils and vegetables [17].

In sub-Saharan Africa, inadequate soil fertility poses a significant impact on farmers, prompting the regular use of chemical and organic fertilizers to enhance soil conditions and yields [18]. In Malawi, over 80% of smallholder farmers rely on manure for agricultural practices despite global evidence linking manure application to ARB dissemination [19]. Most people in urban areas in the country rely on vegetables from informal markets, often sourced from smallholder farms using untreated manure. Nevertheless, the prevalence and transmission pathways of ARBs in Malawian agricultural systems remain poorly understood, threatening both environmental and human health.

Current strategies to mitigate AMR risks, including manure composting and restricted antibiotic use in livestock, face adoption barriers in low-resource settings. Lack of access to composting infrastructure or veterinary oversight incites direct application of raw manure [19,20]. Malawi’s regulatory frameworks for AMR, such as the National Antimicrobial Resistance Strategy 2017–2022, remain under-implemented, particularly in agriculture [21,22]. This gap is alarming given Malawi’s high AMR-associated mortality, with 3600 deaths directly attributable to resistant infections in 2019 [1].

Despite growing recognition of AMR as a One Health priority, limited studies have investigated ARB transmission pathways in Malawi’s manure-dependent farming systems. Previously, scant studies have highlighted the impacts of ARBs in humans and animals [23]. To address this gap, our study aims to characterize the ARB prevalence in poultry and pig manures, agricultural soils, and vegetables in Blantyre city, Malawi. This study provides a critical contribution concerning ARBs and promotes the One Health understanding for sustainable manure management in smallholder farmers.

## 2. Materials and Methods

### 2.1. Study Area

**Figure 1:**
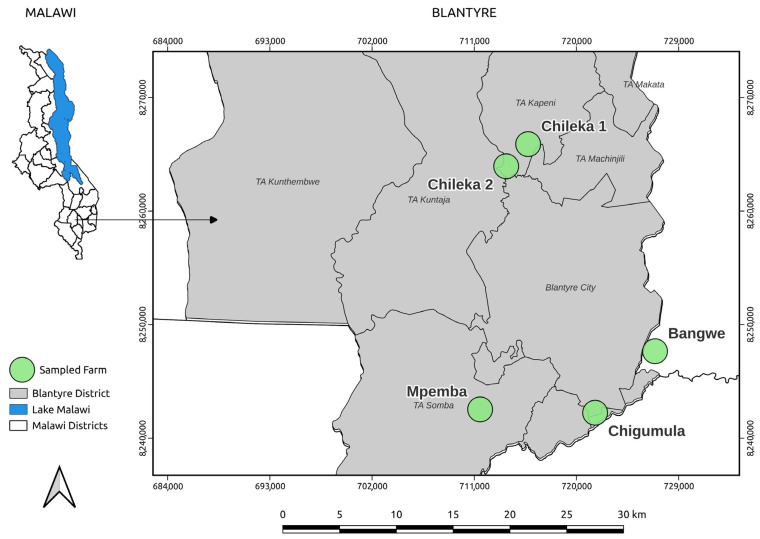
Map of Blantyre City showing farms where samples were collected.

A cross-sectional study was conducted between August and December 2024 in Blantyre, Malawi (15°47′05″ S; 35°00′30″ E), in the area shown in Figure 1. Blantyre’s subtropical climate (annual rainfall: 1127 mm; temperature: 13 to 21 °C) [24] supports smallholder farming. The area has three Extension Planning Areas (EPAs)—Ntonda, Chipande, and Kunthembwe—and there is livestock (poultry/pigs) and vegetable production.

#### 2.1.1. Sample Size and Sampling Strategy

Five farms were purposively selected across the three EPAs (Ntonda, Chipande, and Kunthembwe) to represent typical urban agricultural practices in Blantyre to capture dominant urban farming systems (mixed crop–livestock, variable antibiotic use, and manure management practices). This sample size aligns with recommendations for resource-limited settings where exhaustive sampling is impractical, yet it enables robust cross-farm comparisons of AMR transmission pathways [25,26]. Similar studies in agricultural AMR research have utilized comparable farm numbers: Tadić et al. [27] collected samples from six locations in Spain; Marti et al. [28] and Jauregi et al. [29] used two farms; Yévenes et al. [30] studied seven commercial flocks; and Cobbina et al. [31] and Degaga et al. [32] conducted studies with one and two farms, respectively. Within each farm, composite samples were collected from 7 categories: pig manure, poultry manure, farm soil, home soil, and 3 vegetable species (*Brassica rapa* subsp. *chinensis*, *Brassica rapa*, and *Amaranthus* spp.), resulting in 35 samples (5 farms × 7 categories). The three vegetables were prioritized due to their high consumption raw, differential leaf morphologies influencing bacterial adhesion, and dominance in local markets, factors critical for assessing food safety risks [10,11]. The nested design (with sample categories nested within farms) is consistent with agricultural AMR studies and allows for hierarchical analysis of contamination pathways [26].

#### 2.1.2. Farms Description

Farm 1 (Bangwe) cultivated cabbage (*Brassica oleracea* var. capitata), tomato (*Solanum lycopersicum*), rape (*Brassica rapa*), Chinese cabbage (*Brassica rapa* subsp. chinensis), green pepper (*Capsicum annuum*), onion (*Allium cepa*), spinach (*Spinacia oleracea*), eggplant (*Solanum melongena*), and carrot (*Daucus carota*), with 25 pigs and 250 chickens housed in permanent enclosures (confined housing). Antibiotics administered included triple sulfur (sulfadimidine) and oxytetracycline. Manure was left in the animal pens for three weeks and then composted or applied directly. Farm 2 (Chigumula) grew Chinese cabbage (*Brassica rapa* subsp. chinensis), rape (*Brassica rapa*), onion (*Allium cepa*), sweet potato (*Ipomoea batatas*), and pumpkin leaf (Cucurbita moschata), with 5000 broiler chickens, all housed in closed poultry houses (confined housing). Antibiotic usage featured sulfadiazine and trimethoprim. Manure was stored for 40 days before use. Farm 3 (Mpemba) cultivated rape (*Brassica rapa*), Chinese cabbage (*Brassica rapa* subsp. chinensis), lettuce (Lactuca sativa), spinach (*Spinacia oleracea*), red pepper (*Capsicum annuum*), tomato (*Solanum lycopersicum*), onion (*Allium cepa*), cabbage (*Brassica oleracea* var. capitata), cassava (*Manihot esculenta*), maize (*Zea mays*), banana (*Musa* spp.), sweet potato (*Ipomoea batatas*), and green bean (*Phaseolus vulgaris*), with 200 pigs and 300 chickens, all kept in permanent enclosures (confined housing). Pigs received penicillin, lincomycin, and oxytetracycline; chickens were treated with oxytetracycline, sulfadiazine, and trimethoprim. Manure from chickens was stored for six weeks, and from pigs for four days. Farm 4 (Chileka 1) grew tomato (*Solanum lycopersicum*), cabbage (*Brassica oleracea* var. capitata), green beans (*Phaseolus vulgaris*), maize (*Zea mays*), and Chinese cabbage (*Brassica rapa* subsp. chinensis), with 400 pigs kept in concrete pens (confined housing). Antibiotic regimens included penicillin and oxytetracycline. Manure was stored in a pit for 7 to 10 days and dried. Farm 5 (Chileka 2) cultivated Chinese cabbage (*Brassica rapa* subsp. chinensis), green pepper (*Capsicum annuum*), and rape (*Brassica rapa*), with 84 pigs kept in concrete pens (confined housing) and 150 local (indigenous) chickens raised under free-range management. Antibiotics used were penicillin and oxytetracycline. Manure was heaped without treatment. These farms were selected to capture variability in crop–livestock systems, antibiotic usage patterns, and manure management practices.

### 2.2. Sample Collection

Sample collection methodologies were adapted from established protocols for assessing antimicrobial resistance (AMR) transmission in agricultural systems [28,33] to address the specific context of Malawian urban farming. While Marti et al. [28] and Tien et al. [33] provided foundational approaches for manure, soil, and vegetable sampling in temperate regions, our protocol was optimized for (1) the scale and practices of smallholder urban farms in Blantyre, (2) the commonly consumed Malawian vegetable varieties, and (3) local handling practices including minimal washing of produce. The following subsections detail the specific procedures for each sample type.

#### 2.2.1. Manure Samples Collection

Five manure samples were collected from the five farms (one composite sample per farm). The samples were collected within 2 h of deposition from 10 different points in the livestock pens using a sterile scoop (Alpha Laboratories, Hampshire, UK). These individual samples were stored in a 300 mL labeled sterile sample collection bottle (Alpha Laboratories, Hampshire, UK). The bottle was shaken by hand to create a homogenized composite sample. The samples were immediately placed in a cooler box filled with ice packs and then transported to the laboratory within 1 h of sample collection.

#### 2.2.2. Soil Samples Collection

Five soil samples were collected from vegetable fields and residential areas within a farm (one composite sample of each soil type per farm). For the vegetable fields, soil samples were taken from 10 locations at a depth of 15 cm using a hand auger. A soil sample (300 g) was bulked into a labeled Ziplock bag (S.C. Johnson, Bay, MI, USA) and mixed by hand until homogenous. Similarly, five soil samples from residential areas were collected from walking pathways and places where people frequently stayed (outside the animal pens), using the same protocol as for the vegetable fields. The soil samples were then placed in a cooler box filled with ice packs for transportation to the laboratory within 1 h of sample collection.

#### 2.2.3. Vegetable Samples Collection

Fifteen vegetable samples, specifically *Brassica rapa* subsp. chinensis, *Brassica rapa*, and *Amaranthus* spp., were collected from the five farms. Three samples of each vegetable type were randomly selected from the farms. The whole plant was dug up using a sterilized hand fork, sterilized with 99.9% ethanol (258 Boschkop Road, Mooiplaats, Pretoria, South Africa) between sampling, and soil particles adhering to the roots were carefully removed. The cleaned vegetables were then placed in a clean Ziplock bag (S.C. Johnson, Bay, MI, USA), stored in a cooler box with ice packs, and transported to the laboratory for analysis within 1 h of sample collection.

### 2.3. Sample Preparation

#### 2.3.1. Manure and Soil Sample Preparation

Both manure and soil samples (200 g each) were homogenized with 100 mL laboratory-grade distilled water (VWR, Radnor, PA, USA) and vortex-mixed for 5 min using a Vortex Genie 2 mixer (Scientific Industries, New York, NY, USA) to ensure thorough dispersion of particulate matter and microbial cells. The resulting suspensions underwent sequential filtration: first through sterile sieves (~1 mm pore size) to remove large debris, followed by filtration through 0.45 µm pore membranes (Sterlitech, Auburn, WA, USA) to concentrate culturable bacteria. Final filtrates were collected in sterile 20 mL bottles (Fisher Scientific, Cork, Ireland) for downstream analyses. This protocol follows established environmental microbiology standards, including APHA Standard Methods 9230B [34], and aligns with best practices in antimicrobial resistance studies where sequential filtration improves bacterial recovery and sample quality [35]. It is noted that while 0.45 µm filtration captures most bacteria, some aggregates or biofilms larger than the membrane pores may be underestimated.

#### 2.3.2. Vegetable Samples Preparation

Upon arrival from collection at the farms, roots were washed with distilled water to remove residual soil, and the entire plant was rinsed with 500 mL of distilled water to recover surface-adhered bacteria. The rinse water was homogenized and transferred to sterile bottles for analysis, reflecting typical consumer handling risks. The rinse water was stirred thoroughly and transferred into a 20 mL bottle (Fisher Scientific, Cork, Ireland) for subsequent analysis [28,33].

### 2.4. Bacteria Culture Isolation

Bacterial isolation for all samples was conducted according to Alemi et al. [36]. Filtrates (25 mL) were serially diluted in sterile saline (Alpha Laboratories, Hampshire, UK). Aliquots (1 mL) were pour-plated in triplicate on Nutrient Agar (Oxoid, Hampshire, UK) and incubated for 24 h at 37 °C in a Sanyo MCO-18AIC incubator (Panasonic Healthcare, Tokyo, Japan). Morphologically distinct colonies were subcultured on MacConkey Agar (Oxoid, Hampshire, UK) for Gram-negative selection, Blood Agar (Oxoid, Hampshire, UK) for hemolytic profiling, and Mueller–Hinton Agar (Oxoid, Hampshire, UK) for antimicrobial susceptibility testing preparation. Gram staining was performed using standard protocols with Crystal Violet (Cypress Diagnostics, Hulshout, Belgium), Gram’s Iodine (Cypress Diagnostics, Langdorp, Belgium), Decolorizer (Cypress Diagnostics, Hulshout, Belgium), and Safranin (Cypress Diagnostics, Hulshout, Belgium), with smears examined under a Motic BA310 microscope (Motic, Hong Kong, China). Pure isolates were identified via API 20E test strips (bioMérieux, Lyon, France) incubated for 18 to 24 h at 37 °C, with reactions interpreted per the manufacturer’s coding system. Presumptive identification of non-Enterobacteriaceae organisms (e.g., Pasteurella-like isolates) was based on concordance between API profile, Gram stain morphology, and characteristic colony features on Blood Agar (non-hemolytic growth) and MacConkey Agar (growth inhibition pattern). All microbial culture and enumeration steps were performed in triplicate for each sample to ensure reproducibility of results. The minimum detection threshold was 1 colony-forming unit (CFU) per plate, based on 1 mL aliquots used in pour-plating, and only plates yielding between 30 and 300 CFUs were included for enumeration in accordance with APHA Standard Methods [34].

### 2.5. Antibiotic Susceptibility Tests

Antibiotic susceptibility testing was performed using the Kirby–Bauer disk diffusion method according to the Clinical and Laboratory Standards Institute (CLSI) guidelines (30th edition) [37]. Pure bacterial isolates were suspended in sterile phosphate-buffered saline (PBS) and adjusted to a 0.5 McFarland turbidity standard (~1.5 × 10^8^ CFUs/mL) using McFarland standard solutions (bioMérieux, Lyon Marcy-l’Étoile, France). Within 15 min, a sterile cotton swab was dipped into the suspension and streaked in three directions onto Mueller–Hinton agar plates (Oxoid, Hampshire, UK) to ensure confluent growth. Antibiotic disks (Oxoid, Hampshire, UK) with the following concentrations were applied using a disk dispenser (Oxoid, Hampshire, UK): ampicillin (AMP, 15 µg), anthramycin (ATM, 15 µg) ciprofloxacin (CIP, 5 µg), ceftriaxone (CRO, 5 µg), cefotaxime (CTX, 5 µg), gentamicin (GM, 10 µg), meropenem (MEM, 5 µg), trimethoprim–sulfamethoxazole (SXT, 15 µg), tigecycline (TGC, 15 µg), and vancomycin (VAN, 5 µg). Plates were incubated aerobically at 37 °C for 18 to 24 h. Zones of inhibition were measured to the nearest millimeter using a ruler and interpreted as susceptible, intermediate, or resistant according to CLSI 2023 breakpoints.

### 2.6. Data Analysis

Descriptive statistics, including frequencies and percentages, were used to summarize microbial prevalence across sample types. Composite samples, each representing pooled subsamples from multiple points within a sampling site, were treated as single independent observations in all analyses. Differences in microbial occurrence among farms and between sample types (manure, soil, and vegetable) were assessed using one-way ANOVA, followed by Tukey’s Honest Significant Difference (HSD) tests for post hoc comparisons. Pairwise Jaccard similarity indices were calculated between sample compartments to evaluate microbial community similarity. One-sample *t*-tests were performed to assess whether the observed Jaccard indices differed significantly from zero similarity. A significance level of *p* < 0.05 was used throughout. All statistical analyses were performed using R version 4.5.0 (R Core Team, 2024) within RStudio version 2024.12.0+402.

## 3. Results and Discussion

### 3.1. Bacterial Identification in Samples

#### 3.1.1. Bacterial Identification in Manure

Microbial analysis of manure samples revealed contamination with pathogens. Poultry manure contained *E. coli* in 40% of samples (18/45) and *K. pneumoniae* in 27% (12/27), while pig manure exhibited higher *E. coli* prevalence (52%, 24/46) alongside *K. pneumoniae* (26%, 12/46) and *Acinetobacter* species (13%), as shown in Table 1. Full sensitivity classifications for manure isolates are provided in Appendix A.2 (Table A2) Statistical analysis demonstrated significant farm-level variations in contamination intensity, with the pig manure from Farm 3 showing higher microbial counts than that of Farm 4 (*p* < 0.001), as shown in Table 2. This correlates directly with Farm 3′s practice of applying manure after only 4 days of drying, whereas Farm 4 utilized pit storage (7–10 days) followed by extended drying. The elevated bacterial loads and antimicrobial resistance at Farm 3 could be explained by high animal density and limited biosecurity. Intensive confinement often increases disease burden, prompting more frequent antibiotic use, which selects for resistant bacteria. Poor ventilation, overcrowding, and hygiene challenges inherent in such systems can exacerbate pathogen spread and resistance gene dissemination within farms [38,39]. Similar patterns have been reported in Malawi, where Mankhomwa et al. [40] linked intensive small-scale poultry and pig farming to increased antibiotic use and resistance emergence. Comparable findings from Ethiopia demonstrate that frequent antibiotic administration under intensive husbandry creates selective pressure favoring resistant bacteria in livestock manure [41]. Table 1 shows the microbes that were isolated from the manure samples.

Across the farms, there were observable microbial load differences in pig manure, as shown in Table 2.

The persistence of these pathogens aligns with the study by Jiang et al. [42] that revealed *E. coli* survival for 231 days in manure-amended soils, while Yao et al. [43] observed longer persistence in pig manure compared to poultry waste. These findings are consistent with Ngogang et al. [44], who reported 59.1% *E. coli* prevalence in Cameroonian poultry litter, and Igbinosa et al. [45], who detected ESBL *E. coli* in 84% of manure samples in Nigerian farms, indicating that manure in sub-Saharan Africa frequently harbors antimicrobial-resistant pathogens, comparable to the prevalence rates observed in this study. In Zimbabwe, cloacal swabs from 3050 poultry birds revealed ESBL-producing *E. coli* in 0.7% of flocks and spread across major ESBL genes (CTX-M-14 and CTX-M-79), identifying poultry as reservoirs for multidrug-resistant bacteria [46]. Additionally Hou et al. [47], identified *K. pneumoniae* in 19.9% of livestock manure samples in China.

The high pathogen burden highlights manure’s role as a primary reservoir for antibiotic-resistant bacteria (ARBs), particularly given that 80% of the studied farms applied untreated manure within 10 days of collection. Applying this contaminated manure could facilitate zoonotic transmission of resistance genes to soil microbiomes, ultimately reaching vegetables consumed raw. Farm families face direct exposure during manure handling, while broader communities risk ingestion through contaminated produce, a pathway documented in a study conducted in Ghana, where similar practices led to human ESBL infections [48]. 

#### 3.1.2. Bacterial Identification in Soil Samples

This study revealed distinct bacterial contamination profiles between farm and adjacent home soils. Notably, *K. pneumoniae* prevalence was significantly higher in home soils (67%, 36/54) than in farm soils (28%, 24/85) (*p* < 0.001), with Farm 1′s home soil showing higher microbial counts than other farms (*p* < 0.001). This elevated contamination corresponds to direct manure application without adequate composting.

Contrary, home soil exhibited higher levels of *K. pneumoniae* despite the absence of direct manure application. This pattern can be explained by the environmental persistence and adaptability of *K. pneumoniae*, which can survive extended periods in diverse environments by forming biofilms and resisting antimicrobial treatments [49,50]. Environmental reservoirs of *K. pneumoniae* have been implicated in prolonged outbreaks, with strains persisting for over 20 months in hospital settings, demonstrating their ability to survive on surfaces and in environmental niches [51]. Moreover, *K. pneumoniae* is ubiquitous in soil, water, and vegetation, with environmental isolates sharing genetic and phenotypic traits with clinical strains, indicating potential transmission routes between the environment, animals, and humans [52]. Environmental factors such as climate change may further enhance the bacteria’s survival and dissemination of antimicrobial resistance genes [52]. Similar results were observed in the studies from Burkina Faso for ESBL-producing *K. pneumoniae* and *E. coli*, with reported prevalence ranging from 28 to 36% for *E. coli* and 36 to 62% for *K. Pneumoniae* from agricultural soils [53,54]. Environmental surveillance in Tanzania detected ESBL-producing *E. coli* (29.6%) and *K. pneumoniae* (45.5%) in river water, sediment, and crop soils [55]. Similarly, Igbinosa et al. [45] documented ESBL *E. coli* in 68% of farm soils in Nigeria, demonstrating widespread environmental persistence of resistance traits across different SSA agricultural systems.

In contrast, *E. coli* was absent from home soils, consistent with its generally lower environmental resilience and shorter survival outside host-associated or manure-amended environments [56]. This differential survival suggests that *K. pneumoniae* may serve as a more reliable indicator of long-term environmental contamination, especially in peri-domestic zones, where indirect exposure routes such as dust, wind, and human or animal activity facilitate its spread [57]. Recent genomic surveillance studies emphasize that while *K. pneumoniae* is widely distributed across clinical, animal, and environmental niches, the transmission of multidrug-resistant clones between these compartments remains limited but possible, warranting continued monitoring [58].

Additionally, detecting *Yersinia pestis* in Farm 3 soil (4.7%, 4/85) and *Salmonella choleraesuis* in home soil (11%, 6/54) further highlights the diverse microbial hazards present in these environments. The persistence of *Y. pestis* in soil, often maintained via rodent reservoirs, has been documented by Chomel and Sykes [59], emphasizing potential zoonotic risks in agricultural settings. Additionally, the presence of *Stenotrophomonas maltophilia* (11%, 6/54) in home soils raises concerns due to its intrinsic multidrug resistance and opportunistic pathogenicity, particularly for immunocompromised individuals [60]. Detailed antibiotic sensitivity profiles for these and other soil isolates are provided in Appendix A.2 (Table A2).

Collectively, these findings emphasize soil as a critical reservoir for antibiotic-resistant and pathogenic bacteria within the One Health framework. They highlight the urgent need for improved manure treatment, environmental sanitation, and surveillance to mitigate transmission pathways and protect human and animal health. Table 3 shows the microbes that were isolated from the soil samples.

Observable differences in home soil contamination were found across the farms, as shown in Table 4.

#### 3.1.3. Bacterial Identification in Vegetables

This study’s analysis of surface-adhered bacterial contamination on leafy vegetables revealed distinct crop-specific differences, with contamination patterns reflecting plant morphology, agronomic practices on microbial load, and risks from minimal washing practices. *K. pneumoniae* dominated *Brassica rapa* subsp. *chinensis* (60%, 18/30), *Brassica rapa* (55%, 36/66), and *Amaranthus* spp. (41%, 18/44). *E. coli* was detected in *Amaranthus* spp. (14%, 6/44) and *Brassica rapa* subsp. *chinensis* (20%, 6/30), while *P. aeruginosa* contaminated *Brassica rapa* (7.6%, 5/66) (Table 5). The detection of *K. pneumoniae* and *E. coli* in leafy vegetables is consistent with the results of Diarra et al. [54], who identified ESBL *Klebsiella* spp. in 43.4% and ESBL *E. coli* in 25% of lettuce samples from Burkina Faso, grown under similar agricultural conditions. In South Africa, Richter et al. [61] identified ESBL- and AmpC-producing *Klebsiella* and *E. coli* in 17.4% of vegetables, primarily sold by informal vendors, indicating widespread contamination at the retail level. Similarly, Igbinosa et al. [45] found ESBL *E. coli* in 24.4% of vegetables and 36.6% of market produce in Nigeria, showing high transmission risks from farm to fork. Sore et al. [62] further corroborated these findings, reporting 42.5% ESBL contamination in salad vegetables in Burkina Faso, predominantly *E. coli* and *K. pneumoniae*, reinforcing the Pan-African trend of fresh-produce contamination. Table 5 shows the microbes that were isolated from the vegetable samples.

The absence of *E. coli* in *Brassica rapa* (0%) compared to its presence in *Amaranthus* spp. (14%, 6/44) and *Brassica rapa* subsp. chinensis (20%, 6/30) aligns with studies demonstrating that leaf surface characteristics, such as wax content and topography, influence bacterial adhesion. These results are in line with a study from Tanzania that found *E. coli* on *Amaranthus* spp. and *Brassica rapa* subsp. chinensis samples collected from farm and local markets; however, their study did not include *Brassica rapa* samples [63]. Vegetables with higher epicuticular wax content exhibit smoother surfaces and greater bacterial removal efficacy during washing [64]. The waxy cuticle of *Brassica rapa* may physically impede *E. coli* attachment, as shown by correlations between wax composition (e.g., alkanes and ketones) and reduced bacterial persistence [64]. Additionally, leaf vein density and microtopography factors varying by leaf age and axis affect wash resistance and chlorine survival of *E. coli*, potentially explaining interspecies differences [65]. Similar observations were reported by Truschi et al. [66], who found that baby-leaf salads with higher epicuticular wax content showed significantly lower *Salmonella* attachment. Likewise, Song et al. [67] demonstrated that broccoli cultivars with waxy leaves exhibited reduced *Salmonella* colonization compared to non-waxy variants.

Conversely, *Brassica rapa* harbored a broader spectrum of bacteria, including *K. pneumoniae* (55%, 36/66), *Pseudomonas cepacia* (24%, 16/66), *Erwinia* spp. (7.6%, 5/66), and *P. aeruginosa* (7.6%, 5/66). Both environmental and plant physiological factors may explain this broader microbial presence. The growth habit of *Brassica rapa*, which includes low-lying, soil-contacting leaves, increases its exposure to soil-borne and manure-associated pathogens. Leaf surface microtopography, such as grooves, ridges, and trichomes, can facilitate bacterial retention and protect microbes from desiccation and washing [66]. These structural features may explain why *Brassica rapa* supports a higher microbial load despite lacking *E. coli*.

The adhesion mechanisms of *K. pneumoniae* to leaf surfaces, facilitated by extracellular polysaccharides and biofilm formation, explain its persistence on vegetables [68]. These findings are consistent with studies by Junaid et al. [50], who reported 38% *K. pneumoniae* contamination in Pakistani vegetables, while Falomir et al. [69] isolated multidrug-resistant strains from Spanish produce. The presence of *P. aeruginosa* in consumable crops is particularly concerning given its association with hospital-acquired infections in immunocompromised populations [60].

Importantly, farm management practices, particularly manure handling, were strongly associated with microbial loads in vegetables. As shown in the Tukey test results (Table 6), *Brassica rapa* samples from Farm 3 exhibited significantly higher contamination levels (*p* < 0.001), correlating with fresh, untreated pig manure application. In contrast, Farm 1 utilized composted manure, likely contributing to lower microbial counts. Previous research supports this finding, a study by Zhang et al. [70] demonstrated that anaerobic digestion of pig slurry substantially reduces fecal coliforms in vegetables. Similarly, Atidégla et al. [71] and Machado et al. [72] linked raw poultry and swine manure to increased *E. coli* and coliform contamination in vegetables grown in West Africa and Brazil, respectively. Table 6 presents the results of Tukey’s multiple pairwise comparison (Tukey test) for *Brassica rapa* across different farms, showing the mean differences in values between farm pairs, confidence intervals, adjusted *p*-values, and significance levels.

This study provides the first phenotypic evidence of presumptive *Pasteurella*-like organisms on edible leafy vegetables in Malawi, specifically detected in 45% (20/44) of *Amaranthus* spp. and 6.1% (4/66) of *Brassica rapa* samples. Detailed sensitivity data for all vegetable-associated bacteria are cataloged in Appendix A.2 (Table A2). These organisms, typically associated with animal reservoirs, likely enter the food chain via manure runoff, irrigation water, or direct animal access to fields. *Pasteurella* species are traditionally commensals in the respiratory and gastrointestinal tracts of livestock and birds, including pigs and poultry reared on the studied farms [73,74,75].

*P. multocida* is a well-documented opportunistic pathogen in swine and poultry, responsible for conditions like atrophic rhinitis and fowl cholera [74,75]. Its documented persistence in soil (up to four weeks) and animal remains (several months) supports plausible environmental transmission through manure, aerosols, or irrigation water [74,76].

While not conventional foodborne pathogens, *Pasteurella* species cause zoonotic infections in humans including cellulitis, pneumonia, and bacteremia, primarily through animal bites [77,78]. However, their detection on vegetables raises concern for non-traditional, foodborne, or environmental exposure pathways, especially in settings where close mixing of livestock and crops and minimal biosecurity are common. This suggests that ingestion of contaminated produce could represent an under-recognized route of human infection beyond direct animal contact.

Although typically penicillin susceptible, emerging AMR reports in clinical isolates suggest potential resistance gene transfer risks [74].These emerging resistance traits amplify the public health risk if transmission via fresh produce occurs, particularly affecting vulnerable groups such as the immunocompromised, elderly, and children.

The presence of presumptive *Pasteurella*-like organisms on vegetables from mixed livestock–vegetable farms indicates a potential alternative transmission route in agricultural settings with limited biosecurity. This finding, based on concordant phenotypic characteristics, highlights the need to incorporate fresh produce surveillance into One Health frameworks. Future studies should prioritize molecular confirmation (e.g., 16S rRNA sequencing) to validate these observations and assess genomic AMR determinants. Such investigations will be critical to clarifying this potential foodborne zoonotic threat and supporting integrated AMR and pathogen monitoring efforts.

*Raoultella ornithinolytica* was detected exclusively in *Brassica rapa* subsp. *chinensis* samples, with a prevalence of 20% (6/30), and was absent in *Amaranthus* spp. and *Brassica rapa*. This selective occurrence suggests a crop-specific contamination pathway that may be closely linked to environmental and agronomic conditions unique to this vegetable. Unlike the other crops sampled, *B. rapa* subsp. *chinensis* typically requires high irrigation input and is often grown under conditions that maintain prolonged leaf surface moisture. Such conditions create a favorable microclimate for moisture-adapted bacteria like *R. ornithinolytica*, which is known to thrive in wet environments, including soil, wastewater, and irrigation water [79,80,81]. The organism’s capacity for biofilm formation likely enhances its ability to colonize plant surfaces, making it more resilient to desiccation and washing interventions [82].

Although irrigation water was not sampled in this study, the exclusive presence of *R. ornithinolytica* in this particular crop raises the likelihood that contaminated water sources may have served as the primary transmission route. This is plausible given its established ecology in aquatic systems and its previous detection in vegetables irrigated with surface water in other studies [81]. The lack of detection in soil and other crops further supports the hypothesis of water-mediated contamination rather than soil-originated transmission.

The detection of this bacterium in a raw-consumed leafy vegetable is particularly noteworthy, as *R. ornithinolytica* has been associated with opportunistic human infections, including bacteremia and urinary tract infections, and has shown the ability to acquire multiple antibiotic resistance genes [79]. While its role in foodborne illness is still emerging, its recovery from produce in this study warrants further investigation into irrigation water quality and farm hygiene practices, especially in informal urban agriculture systems, where such monitoring is rarely performed.

In this study, *Erwinia* spp. were detected exclusively in *Brassica rapa* samples (7.6%), with no presence in *Amaranthus* spp. or *Brassica rapa* subsp. *chinensis*. This crop-specific occurrence aligns with the known biology of *Erwinia*, a genus of pectinolytic bacteria responsible for soft rot diseases in various vegetables. These pathogens typically infect through wounds or natural openings in plant tissue and secrete cell wall-degrading enzymes, resulting in tissue maceration and decay [83].

The presence of *Erwinia* spp. in *B. rapa* may be related to its compact leaf structure and soil-contacting base, both of which increase susceptibility to mechanical injury and microbial colonization. Field moisture and harvest handling practices may have further facilitated contamination. Similar findings have been reported in studies from Nigeria and Thailand, where *Erwinia* species were isolated from vegetables using PCR and biochemical techniques [84,85]. These studies emphasize that soft rot-causing bacteria vary across crops and agroecological zones, highlighting the importance of localized surveillance and management strategies [84,85].

While *Erwinia* spp. are primarily known as plant pathogens, occasional reports have noted their isolation from human clinical specimens, although their pathogenicity in humans remains unclear. Nonetheless, their detection in fresh, raw-consumed produce like *B. rapa* underscores the importance of post-harvest hygiene and temperature management, as *Erwinia* thrives under warm, moist storage conditions.

*Pseudomonas cepacia*, now classified under the *Burkholderia cepacia* complex (Bcc), was detected in 24% (16/66) of *Brassica rapa* samples, while *P. aeruginosa* was present in 7.6% (5/66). Both species are known for their ubiquity in soil, water, and plant rhizospheres and their ability to persist under diverse environmental conditions. Their presence on vegetables cultivated in manure-amended soils is not unexpected but raises important implications for food quality and human health.

*B. cepacia* is well documented in agriculture, where it plays a dual role: as a plant pathogen causing diseases such as sour skin in onions [86,87] and as a promising agent for biocontrol and plant growth promotion. Some Bcc strains have demonstrated the ability to suppress fungal pathogens and degrade agricultural chemicals like herbicides and pesticides. This has led to growing interest in their application in sustainable farming systems [88,89]. However, their use in agriculture remains controversial, especially due to their potential to cause opportunistic infections in immunocompromised individuals, such as those with cystic fibrosis [90]. Thus, while Bcc members may offer agronomic benefits, their safety in food systems must be carefully evaluated.

Meanwhile, *P. Aeruginosa,* detected in a subset of the same samples, is a known opportunistic human pathogen with established virulence traits, including biofilm formation, exoenzyme production, and antimicrobial resistance. Studies have repeatedly isolated *P. aeruginosa* from raw vegetables such as lettuce, carrots, and tomatoes [86,91]. Although its antibiotic resistance levels are generally low in foodborne isolates, multidrug-resistant strains have been reported [86]. Importantly, contamination of fresh produce has been linked to intestinal colonization in hospital patients, illustrating a potential pathway for nosocomial outbreaks [86,92]. *P. aeruginosa* isolates from agricultural sources share genetic and functional similarities with clinical strains, suggesting that edible plants may serve as potential transmission sources to humans and animals [93].

Together, the detection of *P. cepacia* and *P. aeruginosa* in *Brassica rapa* underscores two intertwined challenges: the risk of vegetable spoilage and reduced shelf life and the potential transmission of opportunistic pathogens through raw food. While Bcc members may hold promise in sustainable agriculture, their inclusion in the food chain demands robust regulatory oversight and continuous monitoring, particularly in informal farming systems, where composting, water quality, and post-harvest handling remain poorly controlled. Strengthening hygiene practices and integrating these organisms into routine food safety surveillance will be crucial for balancing agricultural productivity with public health protection.

### 3.2. Antibiotic Resistance

#### 3.2.1. Antibiotic Resistance in Chicken and Pig Manures

Livestock manure exhibited universal contamination with antibiotic-resistant bacteria (ARBs) at 100%, with distinct resistance profiles between chicken and pig sources. *K. pneumoniae* isolated from chicken manure showed 100% resistance to AMP, SXT, and GM, consistent with multidrug resistance (Table 7). Aggregate inhibition zone metrics and sensitivity distributions across all antibiotics are summarised in Appendix A.1 (Table A1). *E. coli* from chicken manure exhibited 67% resistance to SXT, 50% to both AMP and cefotaxime (CTX), and 33% to GM while remaining fully susceptible to ciprofloxacin (CIP).

This aligns with studies in Burkina Faso and Nigeria, where poultry manure consistently harbored MDR *K. pneumoniae* with AMP-SXT-GM resistance, a pattern linked to prophylactic antibiotic use in poultry systems [53,94]. The persistence of this resistance phenotype highlights how routine antibiotic exposure selects for conserved resistance mechanisms, as observed in Portuguese farms, where MDR *K. pneumoniae* prevalence reached 90% despite colistin withdrawal [95]. In line with these results, a nationwide survey in Malawi found 44% of *E. coli* isolated from broiler chickens were resistant to CXT, 70% to SXT, and 90% to tetracycline, common drugs used in livestock [96]. Similarly, a nationwide study in Zambia revealed that 86.5% of poultry-derived *E. coli* isolates were resistant to ampicillin, 74.3% to tetracycline, and over 93% displayed multidrug resistance. Whole-genome sequencing confirmed the presence of ESBL-associated genes, along with resistance determinants for quinolones, aminoglycosides, and sulfonamides [97]. This reinforces the notion that local poultry production systems are significant reservoirs of MDR pathogens that can contaminate soils and vegetables through manure use. The detection of MDR *K. pneumoniae* with AMP-SXT-GM resistance profiles in chicken manure is consistent with Kagambèga et al. [53], who identified blaCTX-M genes and multidrug resistance in Enterobacteriaceae from livestock waste in Burkina Faso. Similar patterns were noted by Igbinosa et al. [45] in Nigeria, where 85.9% of isolates from manure exhibited resistance to three or more antimicrobial classes, highlighting the regional scale of resistance dissemination via animal waste. A Zambian study reported 64.6% of *E. coli* isolates from laying hens were MDR, with 30.4% resistant to cefotaxime, 54.6% to tetracycline, and 26.5% to sulfamethoxazole–trimethoprim [98]. In Malawi, small-scale intensive farming practices characterized by close animal confinement and frequent antibiotic use have been identified as key drivers of antimicrobial resistance in livestock [40]. These husbandry factors, especially intensive housing with limited biosecurity, create environments conducive to the maintenance and spread of resistant bacteria, emphasizing the need for improved management practices that reduce disease pressure and antibiotic dependence.

Pig manure, dominated by *E. coli* (44%), exhibited 100% resistance to AMP and vancomycin (VAN), 75% resistance to SXT, and 50% resistance to ceftriaxone (CRO). No resistance was observed to ciprofloxacin (CIP), cefotaxime (CTX), or tigecycline (TGC), while resistance to GM was 25%. This resistance profile suggests potential extended-spectrum β-lactamase (ESBL) production, particularly in relation to the high AMP and CRO resistance rates (Table 7).

These findings mirror reports from Cameroon and Portugal, where pig manure enriched ESBL *E. coli* carrying *blaCTX-M* and *qnrS* genes [44,95]. Notably, the absence of MEM resistance contrasts with intensive Asian systems [99,100] but reflects regional antibiotic usage patterns in sub-Saharan Africa smallholder farms, where access to last-resort antibiotics is limited [41]. The high CTX resistance (50%) observed in our study signals potential ESBL dissemination, corroborated by the predominance of *blaCTX-M* genes in manure-derived *Enterobacteriaceae* in Burkina Faso [53]. Table 7 summarizes the antibiotic resistance profiles of key bacterial species isolated from pig and chicken manure samples, highlighting the percentage resistance (with 95% confidence intervals) to each antibiotic tested and indicating multidrug-resistant (MDR) patterns.

#### 3.2.2. Antibiotic Resistance in Soils

Manure amendment potentially elevated ARB prevalence in agricultural soils, with *K. pneumoniae* isolates from farm soils showing 100% resistance to AMP, SXT, and GM a multidrug resistance (MDR) pattern indicative of strong selection pressure (Table 8 and Table A1). This demonstrates how manure application enriches soil resistomes, as observed in South Africa, where tetracycline resistance genes persisted in manure-amended soils [101]. Manure-amended soils in Burkina Faso were found by Kagambèga et al. [53] to harbor resistant *K. pneumoniae* and *E. coli*, with persistence observed even in treated wastewater irrigation systems. Similarly, in Nigeria, Igbinosa et al. [45] reported that MDR *E. coli* strains were present in 68% of soil samples, supporting the argument that soil serves as a stable reservoir for ARBs following manure exposure.

#### 3.2.3. Antibiotic Resistance in Vegetables

Vegetables universally carried ARBs, with *K. pneumoniae* as the dominant MDR pathogen. Resistance escalation was particularly evident for GM, with vegetable-associated K. pneumoniae showing GM resistance of 67% in Amaranthus spp., 67% in *Brassica rapa* subsp. *chinensis*, and 67% in *Brassica rapa* (Table 9 and Table A1). This consistent pattern suggests environmental selection pressure during plant growth. *Amaranthus* spp. and *Brassica rapa* posed the highest risks, with *E. coli* from *Amaranthus* spp. and *Brassica rapa* subsp. *chinensis* exhibiting 100% resistance to GM, AMP, and SXT, fulfilling MDR criteria. Likewise, *K. pneumoniae* from *Brassica rapa* showed 100% resistance to AMP and high co-resistance to SXT (83%) and GM (67%), indicating 100% MDR (Table 9). These findings align with studies in Ghana and Ethiopia, where manure-fertilized vegetables transmitted MDR *E. coli* and *K. pneumoniae* to consumers [102,103]. In Burkina Faso, Sore et al. [62] found MDR ESBL-producing Enterobacteriaceae in 42.5% of salad vegetables, with resistance profiles including gentamicin, sulfamethoxazole–trimethoprim, and ciprofloxacin. These findings mirror the antibiotic resistance patterns documented in vegetable isolates within this study. Igbinosa et al. [45] reported MDR *E. coli* in 36.6% of Nigerian vegetable isolates, further affirming the role of produce as a key AMR transmission vehicle in sub-Saharan Africa. A related study in South Africa by Richter et al. [61] found that 17.4% of vegetable samples sold at retail contained MDR ESBL/AmpC-producing Enterobacteriaceae, with 96.1% resistant to aminoglycosides and 79.2% testing positive for ESBL activity, illustrating widespread regional parallels in resistance patterns.

Emerging threats include CIP resistance in *Amaranthus* spp. *K. pneumoniae* (39%), including an *E. coli* strain with intermediate resistance, signaling escalating fluoroquinolone risks critical for human enteric infection treatment [104,105]. *Pseudomonas* spp. in *Brassica rapa* samples displayed 100% resistance to SXT, CTX, GM, ATM, and VAN, mirroring MDR patterns in United Arab Emirates vegetables [106]. This resistance continuum from manure to vegetables shows how agricultural practices amplify public health risks when inadequate composting fails to reduce ARB loads [107]. Table 9 presents the antibiotic resistance patterns observed among key bacterial isolates from different vegetable samples. The table shows the percentage resistance (with 95% confidence intervals) for each antibiotic tested, highlights multidrug resistance (MDR) findings, and notes any resistance hotspots by organism and vegetable type.

**Table 7:** Antibiotic resistance in microbes from pig and chicken manures. Values show % resistance (95% CI). MDR: resistance to ≥3 antibiotic classes.

Microorganism	Manure Type	Tests (*n*)	SXT	CIP	TGC	CTX	AMP	GM	MEM	ATM	CRO	VAN	Resistance Pattern
*Acinetobacter* spp.	Chicken	6	100% (21–100)	0% (0–79)	NT	NT	NT	0% (0–79)	NT	0% (0–79)	0% (0–79)	100% (21–100)	SXT-VAN
	Pig	6	100% (21–100)	0% (0–79)	NT	NT	NT	0% (0–79)	NT	NT	100% (21–100)	100% (21–100)	SXT–CRO–VAN
*E. coli*	Chicken	18	67% (21–94)	0% (0–56)	NT	50% (9–91)	50% (9–91)	33% (6–79)	NT	100% (21–100)	NT	100% (21–100)	ATM–VAN
	Pig	24	75% (30–95)	0% (0–49)	0% (0–66)	0% (0–66)	100% (34–100)	25% (5–70)	NT	0% (0–66)	50% (9–91)	100% (34–100)	SXT-AMP–VAN
*K. pneumoniae*	Chicken	12	100% (34–100)	0% (0–66)	50% (9–91)	NT	100% (34–100)	100% (34–100)	NT	NT	NT	NT	SXT–AMP–GM
	Pig	12	100% (34–100)	0% (0–49)	100% (34–100)	NT	100% (34–100)	100% (34–100)	NT	NT	NT	NT	SXT–TGC–AMP–GM

Note: Antibiotic abbreviations are as follows: AMP: ampicillin; GM: gentamicin; CTX: cefotaxime; SXT: trimethoprim–sulfamethoxazole; CIP: ciprofloxacin; TGC: tigecycline; MEM: meropenem; ATM: aztreonam; CRO: ceftriaxone; VAN: vancomycin; and NT: not tested. Multidrug resistance (MDR) was defined as resistance to at least one antimicrobial agent in three or more antibiotic classes, following international guidelines [101].

**Table 8:** Antibiotic resistance in microbes from farm and home soils. Values show % resistance (95% CI). MDR: resistance to ≥3 antibiotic classes.

Microorganism	Soil Type	Tests (*n*)	SXT	CIP	TGC	CTX	AMP	GM	ATM	CRO	VAN	Critical Findings
*E. coli*	Farm	30	60% (23–88)	40% (12–77)	0% (0–49)	75% (30–95)	75% (30–95)	40% (12–77)	0% (0–79)	0% (0–79)	0% (0–79)	AMP-CTX
*K. pneumoniae*	Farm	24	100% (51–100)	25% (5–70)	50% (15–85)	50% (15–85)	100% (51–100)	100% (51–100)	NT	NT	NT	AMP-GM-SXT
	Home	36	100% (61–100)	33% (10–70)	33% (10–70)	0% (0–39)	100% (61–100)	100% (61–100)	NT	NT	NT	AMP-SXT-GM
*Salmonella cholerae*	Home	6	100% (21–100)	0% (0–79)	NT	NT	NT	100% (21–100)	0% (0–79)	0% (0–79)	100% (21–100)	Pan-resistance
*Stenotrophomonas* spp.	Home	12	0% (0–79)	0% (0–79)	NT	NT	NT	0% (0–79)	0% (0–79)	0% (0–79)	100% (21–100)	Carbapenem resistance (VAN)

Note: Antibiotic abbreviations include AMP (ampicillin), GM (gentamicin), CTX (cefotaxime), SXT (trimethoprim–sulfamethoxazole), CIP (ciprofloxacin), TGC (tigecycline), ATM (aztreonam), CRO (ceftriaxone), VAN (vancomycin), and NT (not tested). Multidrug resistance (MDR) was defined as resistance to at least one antimicrobial agent in three or more antibiotic classes, following international guidelines [101].

**Table 9:** Antibiotic resistance in microbes from vegetables. Values show % resistance (95% CI). MDR: resistance to ≥3 antibiotic classes.

Microorganism	Vegetable	Test (*n*)	SXT	CIP	TGC	CTX	AMP	GM	ATM	CRO	VAN	Resistance Hotspot
*E. coli*	*Amaranthus* spp.	6	100% (21–100)	0% (0–79)	0% (0–79)	0% (0–79)	100% (21–100)	100% (21–100)	NT	NT	NT	SXT-AMP-GM
	*Brassica rapa* subsp. *Chinensis*	6	100% (21–100)	0% (0–79)	0% (0–79)	0% (0–79)	100% (21–100)	100% (21–100)	NT	NT	NT	SXT-AMP-GM
*K. pneumoniae*	*Amaranthus* spp.	18	67% (21–94)	33% (6–79)	33% (6–79)	33% (6–79)	100% (44–100)	67% (21–94)	NT	NT	NT	AMP
	*Brassica rapa* subsp. *Chinensis*	18	67% (21–94)	0% (0–56)	33% (6–79)	0% (0–56)	100% (44–100)	67% (21–94)	NT	NT	NT	AMP
	*Brassica rapa*	36	83% (44–97)	33% (10–70)	20% (4–62)	0% (0–43)	100% (57–100)	67% (30–90)	100% (21–100)	0% (0–79)	100% (21–100)	AMP-SXT-GM-VAN
*Pseudomonas* *cepacia*	*Brassica rapa*	26	100% (21–100)	33% (6–79)	NT	100% (34–100)	NT	100% (44–100)	100% (44–100)	0% (0–56)	100% (21–100)	SXT-CTX-GM-ATM-VAN

Note: Antibiotic abbreviations include AMP (ampicillin), GM (gentamicin), CTX (cefotaxime), SXT (trimethoprim–sulfamethoxazole), CIP (ciprofloxacin), TGC (tigecycline), ATM (aztreonam), CRO (ceftriaxone), VAN (vancomycin), and NT (not tested). Multidrug resistance (MDR) was defined as resistance to at least one antimicrobial agent in three or more antibiotic classes, following international guidelines [101].

### 3.3. Microbial Transmission Pathways

**Figure 2:**
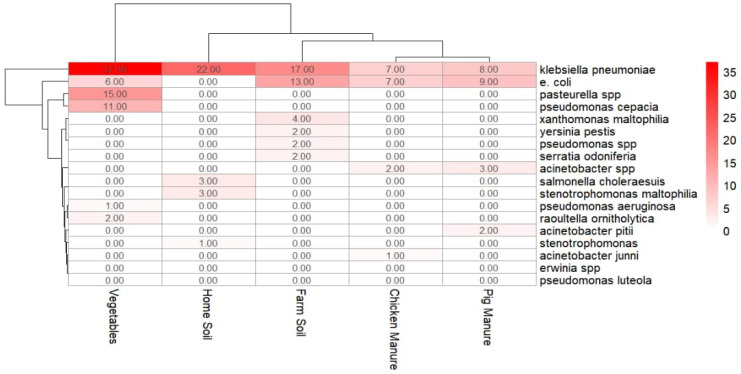
Jaccard similarity analysis of antimicrobial resistance profiles among bacterial isolates across sample compartments.

Microbiological analysis confirmed that *E. coli* and *K. pneumoniae* traversed all agricultural compartments, manure, soils, and vegetables, suggesting potential transmission pathways (Table 10 and Figure 2). While both pathogens demonstrated cross-compartment dissemination, their distribution patterns revealed distinct ecological niches. *E. coli* predominated in livestock-associated environments, whereas *K. pneumoniae* dominated soil and vegetable matrices. This differential distribution suggests distinct ecological adaptations whereby *E. coli* may be more manure associated with limited environmental persistence, while *K. pneumoniae* appears better adapted to soil and plant niches, potentially due to biofilm formation and desiccation resistance mechanisms [95,108,109]. A study by Mwanza et al. [110] in Tanzania similarly demonstrated cross-compartment transmission of antimicrobial-resistant *E. coli* between manure, vegetables, and fish ponds, with ERIC-PCR confirming genetic relatedness of isolates across sources further supporting the integrated pathways observed in this study. In Zimbabwe, pilot data under the WHO ESBL Tricycle initiative found 65% ESBL *E. coli* in poultry and 27% in pregnant women’s rectal swabs, confirming zoonotic and environmental AMR transfer across sectors [111]. Table 10 summarizes the pairwise Jaccard similarity indices for microbial communities detected in manure, soil, and vegetable samples, including confidence intervals, statistical significance, and the interpreted strength of similarity for each comparison.

To quantify the microbial community, i.e., the overlap between compartments, pairwise Jaccard similarity indices were calculated (scale: 0 = no overlap, 1 = identical communities). All comparisons showed statistically significant but weak similarities: manure–soil similarity was 0.247 (95% CI: 0.097–0.390; *p* < 0.05), manure–vegetable similarity measured 0.250 (95% CI: 0.083–0.383; *p* < 0.05), and soil–vegetable connectivity registered 0.287 (95% CI: 0.200–0.407; *p* < 0.05) (Table 10). The strongest connectivity occurred at the soil–vegetable interface, which is consistent with the recognized role of the rhizosphere as a microbial transmission bridge [112]. Notably, the low manure–soil similarity despite direct amendment indicates substantial environmental filtering during manure integration, a phenomenon also reported in other manure-amended agricultural systems [113]. 

Figure 2 illustrates the Jaccard similarity analysis of antimicrobial resistance profiles among bacterial isolates from different sample compartments, providing a comparative overview of shared and distinct resistance patterns across manure, soil, and vegetables.

The observed weak similarities can be attributed to complex ecological dynamics, where environmental filtering through soil properties selectively limited manure-derived taxa, as demonstrated by adsorption mechanisms that reduce *E. coli* transmission in clay-rich soils [108]. Pathogen-specific adaptations further explain differential transmission, with *K. pneumoniae* exhibiting enhanced biofilm formation and phyllosphere persistence [109], while *E. coli* typically shows shorter environmental survival [108,109]. Additionally, the dilution effect from indigenous soil and plant microbiota, as observed in other studies [114,115], contributes to limited taxonomic overlap. Despite the weak community connectivity observed (Jaccard indices: 0.247–0.287), multidrug-resistant *E. coli* and *K. pneumoniae* persisted across all compartments. This suggests that rather than active gene transfer, ecological stability dominates resistance dissemination in these systems. Such persistence may arise from physical co-localization of pathogens in manure-amended soils, enabling plasmid-mediated resistance sharing under spatial constraints [116,117]. Crucially, extended composting (>60 days) disrupts this co-localization by reducing viable pathogen loads, indirectly limiting horizontal gene transfer opportunities [108].

This analysis demonstrates that antimicrobial resistance dissemination operates through partial connectivity rather than complete community transfer. While environmental filters and pathogen adaptations constrain taxonomic overlap, the persistent presence of high-risk multidrug-resistant pathogens confirms an operational farm-to-fork transmission route.

These results suggest that antimicrobial resistance may spread through partial connectivity between farm compartments rather than wholesale transfer of microbial communities. Although environmental conditions and pathogen-specific traits limit bacterial sharing, the persistent contamination of manure, soil, and vegetables with multidrug-resistant pathogens indicates possible farm-to-fork transmission routes. While molecular typing would be required to confirm strain identity across compartments, the presence of four identical species with overlapping resistance profiles suggests environmental dissemination routes. This evidence moves beyond simple “present/absent” frameworks to reveal strategic intervention points. To disrupt transmission, we recommend (1) optimizing manure composting to eliminate pathogen reservoirs, (2) developing soil probiotics to block root-to-plant transfer, and (3) implementing vegetable surface treatments to reduce consumer exposure. These targeted approaches can break critical transmission links while preserving agriculture’s vital role in food security.

### 3.4. One Health Implications: The Silent AMR Crisis on Malawi’s Plate

#### 3.4.1. Human Health Implications

Potential exposure to multidrug-resistant *E. coli* and *K. pneumoniae* from manure to vegetables in Blantyre’s urban farms is a significant public health threat in Malawi. The study found alarming resistance patterns in vegetable-associated isolates, including 100% GM resistance in *E. coli* and 67% in *K. pneumoniae*. This directly undermines the efficacy of GM, a cornerstone of Malawi’s empirical treatment protocols for neonatal sepsis, gonorrhea, and severe Gram-negative infections [118,119,120]. Clinical studies from Queen Elizabeth Central Hospital in Blantyre corroborate this concern, revealing GM resistance in 87.5% of bloodstream *E. coli* isolates between 2018 and 2022 [121].

Equally troubling is the 33% CIP resistance found in *K. pneumoniae* isolated from *Amaranthus* spp., a widely consumed leafy vegetable. CIP remains vital for treating enteric fever, particularly as fluoroquinolone use increases nationally. Recent surveillance indicates a concerning rise of CIP-resistant *Salmonella typhi* in Blantyre, suggesting that resistance may be accelerating under selective pressure [95]. These findings align with national AMR trends showing rising resistance to first-line antibiotics, AMP, sulfamethoxazole–trimethoprim, and CRO among Enterobacterales [122,123].

Most urban consumers in Malawi rely on vegetables from informal markets, often sourced from smallholder farms using untreated manure. Without effective composting and AMR mitigation, this creates a direct ingestion pathway from field to fork. Beyond consumption risks, occupational exposure is an emerging concern. A recent study in Zambia found that poultry farm workers carried *E. coli* strains with 87.9% tetracycline resistance, 48.3% resistance to sulfamethoxazole–trimethoprim, and 46.8% resistance to ampicillin, with nearly one-third (29.3%) classified as MDR and 3.4% as confirmed ESBL producers. This highlights how direct farm contact with contaminated manure can contribute to AMR colonization in humans, particularly workers in informal agricultural settings [124]. Thus, agriculture is no longer a passive contributor but an active amplifier of Malawi’s AMR burden, with vegetables acting as carriers of hospital-relevant pathogens. This reality demands immediate One Health action. Integrated strategies should include extended composting of livestock manure (>60 days), agricultural AMR monitoring, and public health campaigns on food safety. Simultaneously, the Ministry of Health must urgently reassess empirical treatment guidelines, invest in routine microbiological surveillance, and ensure access to newer antibiotics to keep pace with evolving resistance threats. Without such cross-sectoral coordination, Malawi’s food systems may quietly continue to erode the nation’s already fragile therapeutic options.

#### 3.4.2. Environmental Health Implications

The environment has become an entrenched reservoir for antimicrobial resistance (AMR) according to our study findings, driven largely by anthropogenic activities across agricultural, residential, and urban infrastructure domains. Our study detected ESBL-producing *E. coli* in 75% (23/30) (Table 8) of soil isolates from manure-fertilized farms, confirming that manure application transforms agricultural landscapes into long-term reservoirs of resistance genes. These resistant organisms disseminate beyond farms through erosion and irrigation runoff, infiltrating natural water systems. This pattern aligns with previous findings, detecting *blaCTX-M* genes in 68% of Mudi River samples [125]. Notably, our results revealed a 67% (36/54) prevalence of multidrug-resistant (MDR) *K. pneumoniae* in residential soils where no direct manure was applied, implicating broader environmental persistence mechanisms. Passive transport vectors, such as dust, footwear, and animals, play a key role in this dissemination [6].

Environmental vectors such as flies and other arthropods can further contribute to the spread of resistant microorganisms within and between farms, especially under poor housing conditions [39]. Poorly ventilated and overcrowded animal housing facilitates the proliferation of these vectors, which act as mechanical carriers of resistant bacteria. The microbial ecology shaped by these husbandry factors influences the transmission dynamics of AMR bacteria, not only among animals but also to humans through direct contact or via contaminated manure and soils [126]. This amplifies the environmental dissemination pathways beyond direct waste application, highlighting the critical role of housing management in controlling AMR spread.

Beyond agricultural zones, environmental AMR is exacerbated by heavy metals and antibiotic residues in waste streams, which co-select for resistance even in the absence of direct antibiotic exposure [125,127]. This selection pressure promotes horizontal gene transfer among native microbiota, turning soil and water environments into hotspots for resistance evolution. Mwapasa et al. [125] reported ESBL *E. coli* and *K. pneumoniae* in over half of the soil and drainage samples, with peaks during hot–dry and rainy seasons. Urban and peri-urban areas bore the highest burden, driven by inadequate sanitation, stagnant water, and uncontained waste [6,125].

Environmental AMR in Malawi is thus not merely a byproduct of human or animal antibiotic use; it is perpetuated and exacerbated by ecological feedback loops. Contaminated soils, water bodies, and urban infrastructure form an interconnected web of exposure pathways that sustain resistant bacteria within the community, independent of ongoing antimicrobial use. Without environmental surveillance and infrastructural intervention, these systems will continue to function as unmonitored resistance incubators.

#### 3.4.3. Animal Health Implications

Livestock systems in Malawi are central to the persistence and spread of antimicrobial resistance (AMR), shaped by interconnected practices in animal feeding, medication, and waste management. One major pathway involves the use of crop residues and unsold vegetables, often contaminated with multidrug-resistant (MDR) *E. coli* and *K. pneumoniae* as livestock feed. Ingested ARBs establish in the animal gut, where routine antibiotic use further amplifies resistance. This creates a cyclical “farm-to-trough” loop, with manure returning resistant bacteria to crops, water, and feed systems.

Our findings of ESBL-producing *E. coli* and MDR *K. pneumoniae* in manure highlight this problem, with *K. pneumoniae* from chicken manure showing 100% resistance to ampicillin (AMP), sulfamethoxazole–trimethoprim (SXT), and gentamicin (GM) (*n* = 12), and *E. coli* from pig manure exhibiting 100% AMP resistance, 75% SXT resistance, and 33% GM resistance (*n* = 24) (Table 7). This points to declining efficacy of veterinary antibiotics. Although para-veterinarians have demonstrated strong antimicrobial stewardship knowledge [128], farm-level practices like prophylactic antibiotic use and self-medication remain widespread, sustaining resistance selection pressure. Unregulated access to antibiotics exacerbates the issue. In Bvumbwe, livestock farms show high resistance in *S. aureus* to AMP (77.8%) and tetracycline (66.7%), mirroring resistance trends in human clinical isolates [129]. Across the country, resistant *E. coli* has been isolated from broiler chickens, with 44% resistant to CXT and 90% to tetracycline [96]. Small-scale farming, often intensive and poorly regulated, drives misuse; over 53% of broiler farms in Lilongwe reported antibiotic use in their last production cycle, primarily oxytetracycline [5,40].

Without improved veterinary oversight, regulated access, and targeted farmer education, livestock production will continue accelerating resistance emergence. A unified One Health strategy linking agricultural reform, surveillance, and stewardship is urgently needed to preserve antibiotic effectiveness across species.

### 3.5. Study Limitations

While purposive sampling enabled targeted inclusion of diverse farming systems across Blantyre’s EPAs, it may introduce selection bias by under-representing smaller-scale or peri-urban operations. This could limit generalizability to non-commercial urban farms.

Secondly, this study employed the API 20E system for bacterial identification, a practical and widely used biochemical method for Enterobacteriaceae in resource-limited settings. However, API 20E requires pure cultures and is limited to organisms included in its database, which may lead to occasional misidentification or under-detection of fastidious or atypical bacteria in complex environmental samples. Despite these limitations, recent studies from Malawi have successfully applied this methodology in similar contexts, supporting its reliability. Notably, Ibrahim et al. [129] and Banda et al. [23] used comparable biochemical identification methods to characterize antibiotic-resistant *E. coli* and *K. pneumoniae* in wastewater and groundwater in Blantyre and Chikwawa, Malawi, respectively. These precedents reinforce the appropriateness of the current study’s approach while acknowledging that future work could benefit from complementary molecular techniques to enhance identification accuracy.

Thirdly, our vegetable analysis focused on surface-adhered bacteria captured through rinse water, which may underestimate biofilm-associated microorganisms. While this approach accurately reflects consumer exposure risks during typical Malawian minimal-washing practices, it potentially misses biofilm-integrated pathogens that require tissue disruption for detection.

Fourthly, while phenotypic methods such as the disk diffusion technique are standard and widely accepted for antimicrobial susceptibility testing, this study did not include genotypic characterization of resistance genes (e.g., ESBL- or plasmid-mediated resistance determinants). This limits our ability to identify specific genetic mechanisms underlying resistance, including horizontal gene transfer potential. Due to financial and infrastructural constraints, molecular confirmation was not feasible in the current study, but its inclusion is strongly recommended in future research to provide a more comprehensive understanding of AMR pathways in urban agriculture.

Lastly, without genotypic characterization of bacterial isolates, we cannot confirm strain-level transmission across farm compartments. Our pathway analysis should be interpreted as revealing potential rather than confirmed transmission routes.

## 4. Conclusions

This study aimed to determine the prevalence of antimicrobial-resistant bacteria (ARBs) in poultry and pig manures, agricultural soils, and vegetables in Blantyre, Malawi, and to investigate the contamination pathways influenced by smallholder manure management practices. The results revealed that multidrug-resistant *E. coli* and *K. pneumoniae* were widely present across all sample types. These bacteria showed resistance to multiple antibiotic classes, including β-lactams (such as ampicillin and cefotaxime), sulfonamides (e.g., sulfamethoxazole–trimethoprim), and aminoglycosides (such as gentamicin). The widespread multidrug resistance observed highlights the persistence and spread of resistant bacteria in the agricultural environment, independent of specific antibiotic usage patterns.

Although manure amendment potentially facilitated microbial transmission, the overlap of microbial communities between compartments was limited, suggesting that environmental factors and pathogen-specific traits restrict survival and movement across these environments. Current manure management strategies, including composting for ≤6 weeks, proved inadequate to eliminate ARBs, as a significant proportion of vegetable-associated isolates exhibited resistance to multiple antibiotic classes. Notably, soils amended with fresh manure harbored zoonotic pathogens such as *S. choleraesuis* and *Y. pestis*, posing direct risks to farm workers and consumers.

These results highlight a critical trade-off: while manure sustains soil fertility in resource-limited settings, it concurrently propagates antimicrobial resistance (AMR) in agroecosystems. The persistence of resistant microbes, particularly to third-generation cephalosporins, suggests the silent spread of plasmid-mediated resistance mechanisms. Additionally, this study is the first to report on the presence of presumptive *Pasteurella* spp. on edible leafy vegetables, highlighting a novel potential route of zoonotic transmission through produce cultivated on mixed livestock farms.

Future research must quantify resistance gene transfer from vegetables to human microbiomes and assess the health impacts of chronic low-dose exposure from raw produce. Particular urgency should be placed on evaluating fluoroquinolone resistance dynamics given its therapeutic importance. Furthermore, seasonal variation and long-term studies should be considered to enhance our understanding. Additionally, further action such as extending composting time past 60 days, employing vegetable surface hygiene protocols, and advancing irrigation monitoring. As Malawi revises its AMR strategy, this evidence demands coordinated One Health action from farmers adopting validated composting, veterinarians enforcing antibiotic stewardship, and health authorities monitoring environmental hotspots.

## Figures and Tables

**Table 1:** Microbes isolated from manure samples.

Microbe	Chicken Manure (*N* = 45)	Pig Manure (*N* = 46)
*Acinetobacter junii*	4 (8.9%)	0 (0%)
*Acinetobacter pitii*	0 (0%)	4 (8.7%)
*Acinetobacter* spp.	6 (13%)	6 (13%)
*E. coli*	18 (40%)	24 (52%)
*K. pneumoniae*	12 (27%)	12 (26%)
*Serratia odoniferia*	5 (11%)	0 (0%)

**Table 2:** Tukey test results for microbial load differences in pig manure across farms.

Comparison	Difference	Lower CI	Upper CI	Adjusted *p*-Value ^1^
Farm 4 and Farm 3	6.5	3.049183	9.950817	0.006 **
Farm 5 and Farm 4	−6.0	−9.780181	−2.219819	0.011 *

^1^ Significance codes: ** *p* < 0.01; * *p* < 0.05.

**Table 3:** Microbes isolated from soil samples.

Microbe	Farm Soil (*N* = 85)	Home Soil (*N* = 54)
*E. coli*	30 (35%)	0 (0%)
*K. pneumoniae*	24 (28%)	36 (67%)
*Pseudomonas luteola*	5 (5.9%)	0 (0%)
*Pseudomonas* spp.	5 (5.9%)	0 (0%)
*S. choleraesuis*	0 (0%)	6 (11%)
*Serratia odoniferia*	5 (5.9%)	0 (0%)
*S. maltophilia*	0 (0%)	6 (11%)
*Stenotrophomonas* spp.	0 (0%)	6 (11%)
*Xanthomonas maltophilia*	12 (14%)	0 (0%)
*Y. pestis*	4 (4.7%)	0 (0%)

**Table 4:** Tukey test results for home soil contamination differences across farms.

Comparison	Difference	Lower CI	Upper CI	Adjusted *p*-Value ^1^
Farm 3 and Farm 1	−12	−12	−12	<0.001 ***
Farm 4 and Farm 1	−12	−12	−12	<0.001 ***
Farm 5 and Farm 1	−12	−12	−12	<0.001 ***

^1^ Significance codes: *** *p* < 0.001.

**Table 5:** Microbes isolated from vegetable samples.

Microbe	*Amaranthus* spp. (*N* = 44)	*Brassica rapa* subsp. Chinensis (*N* = 30)	*Brassica rapa* (*N* = 66)
*Erwinia* spp.	0 (0%)	0 (0%)	5 (7.6%)
*E. coli*	6 (14%)	6 (20%)	0 (0%)
*K. pneumoniae*	18 (41%)	18 (60%)	36 (55%)
*Pasteurella* spp.	20 (45%)	0 (0%)	4 (6.1%)
*P. aeruginosa*	0 (0%)	0 (0%)	5 (7.6%)
*Pseudomonas cepacian*	0 (0%)	0 (0%)	16 (24%)
*Raoultella ornitholytica*	0 (0%)	6 (20%)	0 (0%)

**Table 6:** Tukey test results for *Brassica rapa* across farms.

Comparison	Difference	Lower CI	Upper CI	Adjusted *p*-Value ^1^
Farm 3 and Farm 1	6.5	2.912001	10.087999	0.004 **
Farm 3 and Farm 2	6.0	2.412001	9.587999	0.006 **
Farm 4 and Farm 3	−6.0	−9.587999	−2.412001	0.006 **
Farm 5 and Farm 3	−6.0	−9.587999	−2.412001	0.006 **

^1^ Significance codes: ** *p* < 0.01.

**Table 10:** Pairwise Jaccard similarity indices of microbial communities in manure, soil, and vegetables.

Comparison	Mean	Lower CI	Upper CI	T-Value	*p*-Value	Strength
Manure–soil	0.247	0.0971	0.39	3.08	0.0185	Weak
Manure–veg	0.25	0.0833	0.383	3	0.02	Weak
Soil–veg	0.287	0.2	0.407	4.84	0.00421	Weak

## Data Availability

The data presented in this study are available from the corresponding authors upon reasonable request.

## Appendix A

### Appendix A.1

**Table A1:** Summary of tests per antibiotic.

Antibiotic	Microbe Diversity *N* = 18 *n* (%)	Number of Tests *N* = 370 *n* (%)	Mean Inhibition Zone	Sensitivity Distribution		
				I	R	S
AMP	4 (22)	39 (10.54)	7.153846	0	37	2
ATM	14 (78)	26 (7.03)	15.576923	0	11	15
CIP	18 (100)	62 (16.76)	25.419355	14	9	39
CRO	15 (83)	27 (7.30)	18.296296	6	5	16
CTX	9 (50)	44 (11.89)	20.613636	0	12	32
GM	18 (100)	65 (17.57	12.307692	1	40	24
MEM	2 (11)	2 (0.54)	24.000000	0	0	2
SXT	12 (67)	53 (14.32)	8.566038	0	43	10
TGC	6 (33)	40 (10.81)	18.925000	1	12	27
VAN	7 (39)	12 (3.24)	2.500000	1	11	0

### Appendix A.2

**Table A2:** Microbe sensitivity across sample types.

Sample Type	Microbe	I	R	S
Chicken manure	*Acinetobacter junni*	1	1	2
	*Acinetobacter* spp.	1	2	3
	*E. coli*	0	7	11
	*K. pneumoniae*	1	7	4
	*Serratia odoniferia*	1	0	4
Farm soil	*E. coli*	1	13	16
	*K. pneumoniae*	2	17	5
	*Pseudomonas luteola*	1	0	4
	*Pseudomonas* spp.	1	2	2
	*Serratia odoniferia*	0	2	3
	*Xanthomonas maltophilia*	1	4	7
	*Y. pestis*	0	2	2
Home soil	*K. pneumoniae*	4	22	10
	*S. choleraesuis*	0	3	3
	*Stenotrophomonas*	0	1	5
	*S. maltophilia*	0	3	3
Pig manure	*Acinetobacter pitii*	1	2	1
	*Acinetobacter* spp.	0	3	3
	*E. coli*	1	9	14
	*K. pneumoniae*	0	8	4
Vegetables	*E. coli*	1	6	5
	*Erwinia* spp.	0	0	5
	*K. pneumoniae*	3	37	32
	*Pasteurella* spp.	2	15	7
	*Pseudomonas aeruginosa*	0	1	4
	*Pseudomonas cepacia*	1	11	4
	*Raoultella ornitholytica*	0	2	4
